# The prognostic value of toxin B and binary toxin in *Clostridioides difficile* infection

**DOI:** 10.1080/19490976.2021.1884516

**Published:** 2021-03-04

**Authors:** Salvador López-Cárdenas, Eva Torres-Martos, Juan Mora-Delgado, Juan Manuel Sánchez-Calvo, Marta Santos-Peña, Ángel Zapata López, María Dolores López-Prieto, Salvador Pérez-Cortés, Juan Carlos Alados

**Affiliations:** Unit of Infectious Diseases and Clinical Microbiology. Jerez De La Frontera University Hospital, Jerez De La Frontera, Cádiz, Spain

**Keywords:** Clostridioides difficile, toxin B, binary toxin

## Abstract

To study the association between detection of the *Clostridioides difficile* gene encoding the binary toxin (CDT) and direct detection of toxinB (TcdB) from feces with the appearance of serious disease, complications, or recurrence in a prospective series of cases. A total of 220 confirmed cases were included, using a two-step algorithm: an initial study to detect the enzyme, glutamate dehydrogenase (GDH), followed, in cases of positivity, by detection of the tcdB. tcdB-positive patients were investigated for the presence of CDT and TcdB. Outcome variables were severe disease, the modified Illinois *C. difficile* infection (CDI) prognostic risk index (ZAR score), the appearance of complications (need for colectomy, CDI-related death, or toxic megacolon) and recurrence. Patients who tested positive for the presence of TcdB in feces were found to have greater disease severity than those who tested negative, with a ZAR score of 35.4% vs. 23% (*p* = .048), a higher recurrence rate (14.6% vs. 5.9%, *p* = .032), and a tendency for higher number of complications (20.7% vs. 11.5%), although without reaching statistical significance (*p* = .053). When presence of CDT was analyzed, higher frequencies of severe disease (39.2% vs. 21.2%, *p* = .005), complications and recurrence (21.6% vs. 10.9%, *p* = .037 and 14.9% vs. 5.8%, *p* = .029; respectively) were observed in patients where CDT was detected. TcdB and CDT act as prognostic markers of the appearance of serious disease, complications or recurrence in cases of CDI. Simultaneous detection of both markers, TcdB and CDT, had a greater impact on the prognosis than when they were detected separately.

## Introduction

*Clostridioides difficile* infection (CDI) is one of the most frequent causes of infection associated with the healthcare environment, responsible, according to some series, for up to 1% of annual hospitalizations.^1^ In the last two decades, the incidence of this entity has increased alarmingly by up to 200%.^1^ In Spain, the incidence rose from 3.9 to 12.2 cases per 10,000 hospitalizations during the period 1999–2007.^2^ The current average incidence of CDI in Spain is estimated at 17.1 cases per 10,000 hospital admissions, range 12.2–24.0 cases per 10,000 hospital admissions.^3^ The increased annual incidence leads to an increase in the direct and indirect costs associated with this entity^4^

Despite this increase, the number of CDI diagnoses may be underestimated^5^The introduction into laboratories of molecular diagnostic techniques for CDI detection that are more sensitive than those previously available^6^has contributed to an increase, of up to 100% in some contexts, in the number of cases detected. This has raised concern in the scientific community about the possible overdiagnosis of this pathology^7–9^

Hence, the application of molecular techniques with high diagnostic sensitivity has often led to clinic-microbiological dissociation^9^which makes it even more necessary to determine which factors are associated with or intrinsic to the appearance of serious episodes and/or with a high risk of recurrence. The main risk factors for recurrence described so far are: advanced age, concomitant use of antimicrobials, parenteral nutrition, use of proton pump inhibitors, previous episodes of CDI, certain hypervirulent strains of *C. difficile*, immunosuppression, high burden of toxigenic *C. difficile* in stools, direct detection of toxin in stools, and delayed resolution of first-episode symptoms. A multitude of prediction models of recurrence based on scoring systems have been developed, but none has been sufficiently sensitive and specific for general use^10–14^There is no consensus about direct detection of the toxin in stools as a risk factor for recurrence, and contradictory studies have been published.^8,15^

The application of molecular methods has facilitated the search for other genes associated with virulence, such as the gene encoding the binary toxin. This toxin has also been proposed as a risk factor for the onset of recurrence, complications, and serious infections, although, as with the direct toxin, the studies are contradictory^16–18^

In order to contribute to a better clinical-microbiological association for the treatment of CDI, the objective of this study was to analyze the correlation between two biomarkers, the presence of toxin B in feces (TcdB) and identification of the gene encoding the binary toxin (CDT), with the appearance of complications, serious illness, and/or recurrence.

## Methods

Study carried out in a second-level hospital, a reference for a population of about 350,000 inhabitants. For oncohematological diseases, the population cared for is about 450,000 inhabitants. Our sample of patients corresponded to inpatients and outpatients diagnosed in the Microbiology Laboratory of our hospital.

This was a prospective study conducted between January 2012 and September 2018, after completing the recruitment pre-established by the design of the study. The sample size estimation was performed using the EPIDAT 4.1 package, assuming a proportion of severe disease in 30% of patients with a diagnosis of CDI, for a precision of 7% and a confidence level of 95%. Included were all patients seen with diarrhea lasting more than 72 hours with a microbiological diagnosis of CDI. All included cases were first episodes. The study was approved by the Ethics committee of the Center of Jerez University Hospital.

For the microbiological diagnosis, a two-step algorithm was followed: first, feces were tested for detection of the enzyme, glutamate dehydrogenase (GDH) (Health & Research C difficile GDH, VEGAL FARMACEUTICA SL, Spain), followed, in case of positivity, by a nucleic acid amplification test (PCR) to detect the presence of the tcdB gene encoding toxin B (tcdB) and the cdtA gene of the binary toxin (CDT) (GenXpert® C. difficile BT, Cepheid Iberia SLU, Spain). The laboratory testing scheme for CDI was in place and unchanged for the entire period of the study. The GenXpert® test is capable of detecting a deletion at nucleotide 117 (tcdCΔ117) compatible with ribotype 027 strains. The higher toxin production of the 027 strains is attributed to deletions of the tdcC regulatory gene, which is why we detect these strains with greater virulence. In addition to the samples with a confirmed diagnosis, stool samples were also investigated for the presence of TcdB by a direct toxin test (Uni-Gold®C). Trinity Biotech Uni-Gold®*C. difficile* Toxin A/B (Farnsworth Ct Carlsbad. California USA) is a rapid lateral flow immunoassay for the qualitative detection of *C. difficile* toxins A and B in stool (Algorithm 1 and Table 1).

**Table 1.** Evolution of stool samples

	2012	2013	2014	2015	2016	2017	2018	Total
GDH	347	360	396	608	751	745	556	3763
GDH+	42	54	41	86	76	85	61	445
tcdB+	21	26	21	45	38	42	27	220
Recurrence	3	1	0	1	2	9	3	19
IR nosocomial	7,8	8,9	7,3	15	11	9,8	11,6	

IR: Incidence rate (x10.000 admissions)

Clinical, analytics, epidemiological, and treatments variables were also collected from the cases.

“Case” was defined as the presence of diarrhea for 72 hours or more, together with detection of tcdB.

The criterion to establish the “nosocomial” origin of CDI was temporary. Nosocomial acquisition was considered if symptoms appeared 48 h after hospital admission or before 7 days after discharge from hospital.

Cases associated with health care were defined as those that occurred in patients with regular contact with hospital environment (day hospital, minor surgical procedures) without criteria of nosocomial origin.

We created a variable conjugated with TcdB and CDT, in such a way that we obtained four possible categories to assess their independence.

“Recurrent CDI” was considered when the reappearance of symptoms occurred within 8 weeks after the onset of a previous CDI episode, provided that the symptoms of the previous episode had resolved after completion of the initial treatment.

We chose the ZAR score to define severe disease after reviewing the literature available at the beginning of the study (2011). Severe disease was established when 2 or more criteria of the ZAR severity assessment score were met, in accordance with the clinical trial carried out by Zar et al.^19^ One point was given for each of the following: age>60 years; temperature>38.3°C; albumin <2.5 mg/dL; leukocytosis>15,000 cells/mm^3^. Two points were given for endoscopic evidence of pseudomembranous colitis and treatment in the ICU.

Registered complications were the presence of toxic megacolon, need for colectomy, and CDI-related death.

Death related to CDI was attributed in the absence of other concomitant entities that would justify the death of the patient.

The patients were followed up at 90 days with the purpose of establishing recurrences or fatal outcome.

## Statistical analysis

Univariate/multivariate association analysis was performed. Statistical significance was calculated using the Student’s t-test for continuous or quantitative variables and the chi-squared or Fisher’s exact test for qualitative variables. SPSS v.20 was used for the analysis.

## Results

Descriptive analysis

Two hundred and twenty patients were included in the sample; 113 (51.4%) were women and median age was 70 years old (range 14–96). The majority were admitted patients (n = 194; 88.2%) and most cases (n = 83; 37.7%) occurred in the internal medicine/infectious diseases unit (Table 2).

**Table 2.** Source by clinical unit of admitted patients

Clinical Unit	n	%
Internal Medicine	48	21.8
Infectious Diseases	35	15.9
Hematology	40	18.2
Oncology	29	13.2
Digestive System	40	18.2
General Surgery	5	2.3
Nephrology	9	4.1
Intensive Care Unit	5	2.3
Neurology	4	1.8
Orthopedics	1	0.5
Emergencies	3	1.4
Urology	1	0.5

Clinical characteristics and comorbidities

Table 3 shows the comorbidities of the studied population.

**Table 3.** Comorbidities

Comorbidities (N = 220)	n	%
Systemic arterial hypertension	106	48,2
Diabetes mellitus	63	28.6
Obesity	19	8.6
Cirrhosis	12	5.5
Chronic obstructive pulmonary disease	20	9.1
Cardiopathy	57	25.9
Chronic kidney disease	42	19.1
Dialysis	13	5.9
Previous abdominal surgery	11	5
Neoplasm	94	42.7
Immunosuppression	91	41.4
Transplant	22	10
Bowel disease	44	20
Inflammatory bowel disease	16	7.3
Diverticulosis	16	7.3
Charlson index: scale 0–4.0 (Mean (SD))	3.45 (2.34)	
McCabe: non-fatal disease	119	54.1
McCabe: ultimately fatal disease	78	35.4
McCabe: rapidly fatal disease	23	10.4

In 92.7% of the cases, patients had some comorbidity. The level of comorbidity among patients was low (Charlson Index 3.45 ± 2.34). Almost half (42.7%; n = 94) had some type of neoplasia and 41.4% (n = 91) were immunosuppressed. This high percentage of patients with neoplasia would be justified by the type of patients we attend. Sixty-one cases had severe disease (27.7%) according to the ZAR index. Thirty-two patients (14.5%) presented complications, and a small number were fatal (n = 9; 4.1%). All-cause mortality was 9.1% (n = 20). Recurrence was observed in 20 (9.1%) patients. Only one patient required colectomy as a result (0.5%) and 3 (1.5%) were admitted to the ICU.

Two hundred and nine patients (95%) had been previously treated with antibiotics, and the ones most frequently used were cephalosporins (n = 62; 28.2%), penicillins (n = 61; 27.7%), carbapenems (n = 49; 22.3%) and quinolones (n = 45; 20.5%). In almost half the patients, antibiotic therapy was suspended at the time of the diagnosis, and in 62 (22.8%), it had already been suspended before the episode.

Most cases were nosocomial (n = 133; 60.5%) but it is worthy of note that 49 (22.3%) were community-acquired. There were 38 (17.8%) cases related to healthcare. All patients had diarrhea, but only 29 (13.2%) had more than 10 stools/day; 37 (16.8%) had abdominal pain and 33 (15.4%) had higher creatinine than their baseline.

Microbiological diagnosis

Two hundred and seventeen of the initial 220 patients were tested for the presence of TcdB in feces, with positive results in 82 (37.8%). The presence of CDT was investigated in 211 patients and was detected in 74 (35.1%).

Both markers were present in 33 (15.9%) cases, and neither of them was detected in 97 (46.6%) patients (Table 4).

**Table 4.** Presence of biomarkers

Biomarkers	Category	n	%
TcdB (n = 217)	Positive Negative	82 135	37.8 62.2
CDT (n = 211)	Positive Negative	74 137	35.1 64.9
TcdB and CDT (n = 208)	B Toxin + and Binary Toxin +	33	15.9
	B Toxin + and Binary Toxin -	40	19.2
	B Toxin – and Binary Toxin +	38	18.3
	B Toxin – and Binary Toxin -	97	46.6
TcdB: Toxin B; CDT: gene encoding binary toxin			

Patients detected with TcdB in feces had greater clinical severity and more recurrences (*p* = .048 and *p* = .032, respectively). These patients had more complications, although without reaching statistical significance (*p* = .053) (Table 5).

**Table 5.** Relationship between TcdB/CDT and severe disease, complications and recurrence

		TcdB			CDT			TcdB/CDT				
		TcdB+	TcdB-	p-value	CDT+	CDT-	p-value	TcdB+/CDT+	TcdB-/CDT+	TcdB+/CDT-	TcdB-/CDT-	p-value
Severe disease^c^ n (%)	Yes	29 (35.4)	31^20^	0.048^a^	29 (39.2)	29 (21.2)	0.005^a^	15 (45.5)	13 (34.2)	11 (27.5)	18 (18.6)	0.018^a^
	No	82 (64.5)	135 (77)		74 (60.8)	137 (78.8)		18 (54.5)	25 (65.8)	29 (72.5)	79 (81.4)	
Complications n (%)	Yes	17 (20.7)	15 (11.5)	0.053^a^	16 (21.6)	15 (10.9)	0.037^a^	11 (33.3)	5 (13.2)	5 (12.5)	10 (10.3)	0.013^a^
	No	82 (79.3)	135 (88.5)		74 (78.4)	137 (89.1)		22 (66.7)	33 (86.8)	35 (87.5)	87 (89.7)	
Recurrence n (%)	Yes	12 (14.6)	8 (5.9)	0.032^a^	11 (14.9)	8 (5.8)	0.028^a^	8 (24.2)	3 (7.9)	3 (7.5)	5 (5.2)	0.031^b^
	No	82 (85.4)	135 (94.1)		74 (85.1)	137 (94.2)		25 (75.8)	35 (92.1)	37 (92.5)	92 (94.8)	

^a^Pearson’s Chi-square; ^b^Likelihood ratio; ^c^ ZAR score

When CDT was analyzed, a similar pattern to that of toxin B was observed, although with a greater difference between groups in the context of severe disease (39.2% vs. 21.2%, *p* = .005). A higher percentage of patients with CDT also presented complications and recurrences (*p* = .037 and *p* = .029, respectively).

We only obtained one case of CDI per ribotype 027 strain, therefore associations between groups have not been studied.

The simultaneous detection of both markers, TcdB and CDT, had a greater impact on the prognosis than when they were detected separately. Thus, the simultaneous presence of TcdB and CDT was associated with more cases of severe disease, complications, and recurrences, compared to when only one of them was detected. When analyzed independently, it was observed that the presence of CDT involved a higher percentage of patients with severe disease, complications, and recurrence than when TcdB was detected in isolation (Table 5).

## Discussion

At the beginning of the study, the role of TcdB as a prognostic value was uncertain. In recent years, several studies, including ours presented here, have established a clear role for TcdB as a predictive risk factor for the appearance of severe disease, recurrence, and complications related to CDI, and this relationship with TcdB^9,16,20–22^ is becoming increasingly. The same have happened with the presence of CDT, although there is still little evidence to support its use as a prognostic factor.^16–18^ In our case, we demonstrated that the detection of CDT also presents a greater risk of serious disease, complications, and recurrences.

It is striking how the number of samples has increased over the years studied, which is related to a higher degree of suspicion after an increase in the sensitivity of diagnostic techniques. This increase was evident since the first months following the utilization of these new techniques. Before these new techniques were established, there was a lower rate of clinical test prescriptions for CDI by the physicians, due to the low sensitivity of the previously available techniques (TcdB). The incidence has increased progressively over the years of study, with the presence of a peak in 2015, coinciding with a nosocomial outbreak in the ward of oncohematological patients. Similarly, the number of recurrences that occurred in 2017 should be highlighted, without finding any factor that justifies it. (Table 1). The high percentage of patients with CDI and neoplasms may be due to the fact that we are in an area of high incidence for neoplastic processes compared to other areas of the Andalusia, in addition to the characteristics of our hospital.^23^

The objective of this study was to provide more information for decision-making in the initial management of patients with CDI. The constant increase in cases of CDI, the latest treatment recommendations provided by the different Medical Societies, and the availability of new therapeutic strategies make essential to have strong criteria to predict severe CDI, risk of recurrence, or complications. In our series, it was observed that those patients with the toxin detected directly from feces samples presented greater clinical severity than those where the toxin was not detected. Certain studies consider that the presence of TcdB in feces is necessary for diagnosis of CDI, with cases diagnosed by detection of tcdB with molecular techniques and in the absence of TcdB being classified as of lesser severity or simply colonization with C. difficile.^6,9^ The presence of severe disease in TcdB- patients could be explained by a low toxin load in the stool or degradation of the toxin due to delays in the transportation of samples;^21^ on such occasions, repeat testing for detection of TcdB is advisable in order to increase the sensitivity of the test.^21^ *Polage et a*l^9^ suggest that over diagnosis and overtreatment may be due to using molecular techniques as the only diagnostic criterion. Other studies report low mortality in the TcdB-group of patients, which is why the treatment of such cases is questioned. The fundamental limitation of these studies is that they are short series with a low number of CDI-associated deaths, which makes this hypothesis of little value, as it does not reach statistical significance.^7,8,20,22,24^

However, a different study^9,15^ associates the presence of TcdB+ and tcdB+ with higher percentages of severe disease, recurrence, and complications.

In our series, in the TcdB-group, almost a quarter of patients (23%) had severe CDI, 11.5% had some type of complication, and 5.9% had at least one recurrence. TcdB-present lower risk of serious disease, complications, or recurrences, but they are not exempt, so each case should be evaluated individually in order to establish the necessity of treatment and best possible therapy. Similar findings have also been described by *Guerrero et al*^25^ and *Lashner et a*l,^26^ who did not find significant differences between TcdB + and TcdB-patients in the appearance of recurrence and severe disease.

Other studies support the performance of molecular techniques that include determination of CDT, since the presence of this gene is associated with an increased risk of recurrence and disease severity.^16–18^ In addition, *Barbut et al*^16^ report increased mortality (RR 2.55, 95% CI 1.25–5.21) as well as severe CDI (RR 3.38, 95% CI 1.29–8.85) in CDT+ patients. Meanwhile, *Bacci et al*^17^ found that mortality was higher in the CDT+/TcdB+ group than in the CDT-/TcdB+ group (RR 1.8, 95% CI 1.2–2.7). This trend was also detected in our study, with a higher rate of complications, recurrence, and severe disease in CDT+ patients than in CDT- patients, regardless of whether TcdB was present or not.

To date, the combined variable, CDT/TcdB, has not been reported in the literature as a risk factor for the appearance of recurrence, severe disease, or complications. 45.5% of CDT+/TcdB+ cases presented severe disease compared with 18.6% in the CDT-/TcdB-group (*p* = .018). This association in those with CDT+/TcdB+ was similarly repeated in terms of complications (33.3% vs 10.3%, *p* = .013) and recurrence (24.2% vs 5.2%, *p* = .031).

The recurrence rate in our center is 9.1%, much lower than the 18.3% established by the Centers for Diseases Control and Prevention (CDC).^27^ Spanish series put the percentage of recurrence at around 12–18%.^9,10^ In 2018, the IDSA updated the treatment for CDI and restricted use of metronidazole to very few circumstances. Two randomized clinical trials conducted in the 1980s and 1990s comparing metronidazole versus vancomycin therapy found no differences in results, although each study group included fewer than 50 patients.^28^ Since 2000, however, randomized placebo-controlled trials have shown that oral vancomycin is superior to metronidazole for clinical cure but there is no difference in recurrence rates between the two drugs.^27,28^

Other drugs such as fidaxomicin and bezlotoxumab have been shown to reduce the incidence of recurrences.^29–31^ In conclusion, adding CDT and TcdB detection to the diagnostic algorithm for CDI after confirming the presence of the tcdB of *C. difficile* by a molecular technique, is useful for early detection of patients at higher risk for complications, severe disease, or recurrence. In cases with TcdB +/CDT + we suggest early treatment and avoid recurrences as far as possible, since we currently have treatment for this purpose (fidaxomicin and bezlotoxumab). Similarly, in severe disease with TcdB +/CDT +, the use of drugs that decrease the risk of recurrence (fidaxomicin or vancomycin + bexlotoxumab) and shorten the duration of symptoms (vancomycin and fidaxomicin vs metronidazole) would be indicated. All these hypotheses should be tested in subsequent studies.

**Algorithm 1:**
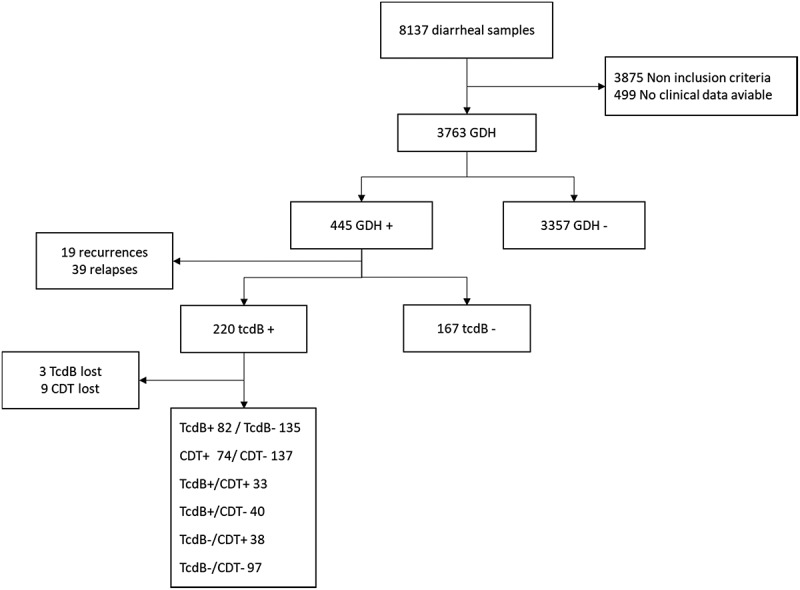
Patient selection scheme

## Discosure

All authors report no conﬂicts of interest relevant

to this article.
